# Sample Preparation and Liquid Chromatography Mass Spectrometry Analysis of Alkylphenolic Compounds and Steroid Sex Hormones in Sediments

**DOI:** 10.1100/tsw.2002.351

**Published:** 2002-06-12

**Authors:** Mira Petrovia, Damia Barcela

**Affiliations:** Department of Environmental Chemistry, IIQAB-CSIC, Jordi Girona 18-26, Barcelona, Spain

**Keywords:** restricted access material (RAM), column switching, endocrine-disrupting compounds, sediments, accelerated liquid extraction

## Abstract

A new methodology, based on the use of accelerated solvent extraction (ASE) and highly selective cleanup using restricted access material (RAM) on-line coupled with liquid chromatography–mass spectrometry (LC–MS), is presented for the simultaneous and unequivocal determination of alkylphenol ethoxylates (APEOs), their degradation products and halogenated derivatives, and steroid sex hormones in sediment samples. Using the integrated RAM–LC–MS system, the simultaneous determination of alkylphenolic compounds and sex hormones was achieved, yielding recoveries higher than 60% and producing low MS background noise.

## INTRODUCTION

Contaminated sediment, especially sludge produced by sewage treatment plants (STPs), can be considered as one of the most complex matrices to be analyzed. In order to isolate endocrine-disrupting compounds (EDCs) from the solid matrix and to achieve the low limits of detection required, substantial analyte enrichment is necessary. Different extraction protocols, employing Soxhlet extraction, sonication, supercritical fluid extraction (SFE), accelerated solvent extraction (ASE), or microwave-assisted extraction (MAE), have been developed[1]. Because of the complexity of samples, a substantial amount of interfering substances are found in crude extracts. The cleanup step aimed at the reduction of the matrix content is generally based either on solid-phase extraction (SPE) or on solid–liquid adsorption chromatography in open columns using a combination of different adsorbents. However, high matrix load affects the performance of the extraction sorbent and gives rise to the partial coextraction of interfering substances.

Among the different sample preparation techniques available, an integrated column-switching system, in which a precolumn filled with restricted access material (RAM) is coupled to the analytical liquid chromatography (LC) column via a switching valve, offers fully automated on-line enrichment of analytes and sample cleanup[2,3,4,5]. In RAM–LC integrated systems, the analyte fraction is selectively extracted and enriched at the stationary phase, while the matrix components are flushed into waste. The porous LiChrospher RP–ADS RAM precolumns (Merck, Darmstadt, Germany), consisting of a hydrophilic and an electroneutral external particle surface (alkyl-diol silica) and a hydrophobic reversed-phase internal surface (C4, C8, or C18), are specially designed for the direct and repetitive injection of samples. The bimodal properties allow retention of low molecular analytes at the lipophilic pore surface, while macromolecular constituents are excluded (cut-off 15 kDa). After the enrichment and cleanup step, the analyte fraction is transferred in the back-flush mode from the precolumn onto an analytical column. All transfer and elution processes and the conditioning of both columns are performed with continuously pumping devices, resulting in a fully automated system and high sample throughput.

The multidimensional LC combined with an effective extraction using ASE saves hours of sample preparation in comparison with conventional sample preparation techniques (Fig. 1) and gives improved results. However, in order to achieve good precision, accuracy, and sensitivity of determination, the operating parameters should be carefully optimized. These include the selection of a sorbent material (C4, C8, or C18), the selection of appropriate mobile phases, for both, the elution of matrix components and for the transfer of analyte onto LC column, and the switching time as defined by the matrix elution profile, breakthrough time of analytes, and analyte elution profile.

This study focused on the determination of two groups of EDCs. The first included alkylphenolic compounds: nonyl- and octylphenol ethoxylates (NPEOs and OPEOs), nonyl- and octylphenoxy carboxylates ($NP_1ECs$ and $OP_1ECs$), and nonyl- and octylphenol (NP and OP) and corresponding halogenated (chlorinated and brominated) derivatives (XAPEOs, XAPECs, and XAPs). The second group included the natural estrogen estradiol (E2) and its main metabolites estriol (E3) and estrone (E1), the synthetic estrogens ethynyl estradiol (EE) and diethylstilbestrol (DES), the natural hormone progesterone (PROG), and the synthetic progestogens norethindrone (NOR) and levonorgestrel (LEV).

## EXPERIMENTAL METHODS

### Accelerated Solvent Extraction of Solid Samples

Extractions were carried out using a Dionex ASE 200 (Dionex, Idstein, Germany). For extraction, 5.00 g of freeze-dried sediment was mixed thoroughly with $Na_2SO_4$ and filled into 11-ml stainless-steel extraction cells. Different solvent mixtures and temperatures were tested, and optimized conditions were as follows: a mixture of acetone/methanol (1:1, v/v) was used as the extraction solvent, with temperature 50$^o$C, pressure 1500 psi, heating time 5 min, and two 5-min cycles of static extraction. As a final step, the cell was purged with gaseous nitrogen. The total

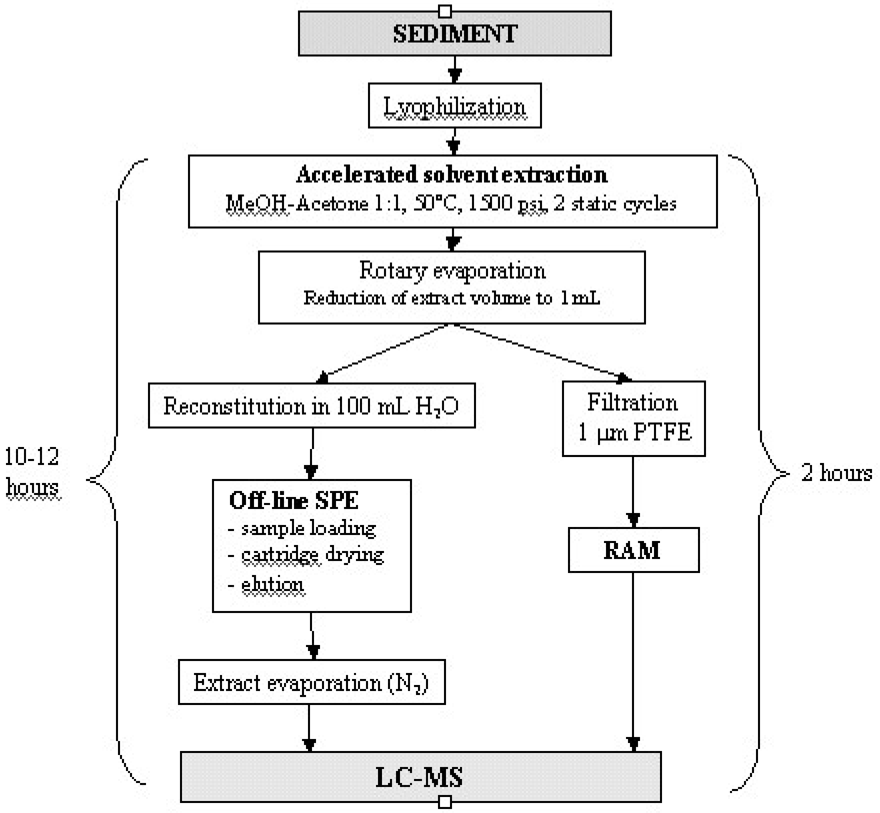

**FIGURE 1**. Conventional sample preparation vs. RAM–LC integrated method.

volume of extract was ~20 ml. The extracts were concentrated to an approximate volume of 1 ml using a rotary vacuum evaporator at 30°C and redissolved in 100 ml of HPLC water. Subsequent cleanup of extracts was performed by SPE using LiChrolute C18 cartridges (Merck, Darmstadt, Germany) as described elsewhere[6]. All experiments were performed with river sediment spiked with 100 ng/g of the composite standard solution of alkylphenolic compounds and steroid sex hormones 72 h before analysis.

## RAM–LC Column Switching

The column-switching program is shown in Table 1. The first step was a fractionation step. The sample was applied via an autosampler to the RAM column and flushed using the mobile phase delivered by an HP 1090 pump. During this step, the analytes were retained on the RAM precolumn, while matrix components were flushed into the waste. Step 2 was the transfer of analytes from the RAM column onto the analytical column using the mobile phase with the stronger elution power, delivered by pump 1. The third step was separation performed in a conventional manner.

**TABLE 1**
**Column Switching Program**

| Pump 1 (Merck LC 6200A) | | | Pump 2 (HP 1090) | | |
|---|---|---|---|---|---|
| 0–4.9 min | $H_2O$:ACN 70:30 | Analytical column conditioning | 0–5 min | $H_2O$:ACN 95:5 | Sample loading into RAM |
| 5–8.9 min | $H_2O$:ACN 70:30 | Analyte transfer to AC | 5–40 min | $H_2O$ + 0.5% HCOOH ACN | RAM column cleaning |
| 9–45 min | Gradient elution | LC separation | 40–45 min | $H_2O$:ACN 95:5 | RAM column conditioning |

## Mass Spectrometric Detection

Detection was carried out using an HP 1040 M diode array UV–Vis detector coupled in series with an LC–MSD HP 1100 mass selective detector equipped with an atmospheric pressure ionization source and electrospray (ESI) interface. The operating parameters were as follows (positive ionization mode / negative ionization mode): drying gas flow 12/11 l/min; drying gas temperature 375/325°C; nebulizer pressure 55/60 psi; capillary voltage 3500/5000 V and fragmentation voltage 60/100 V.

Diagnostic ions used for the analysis of APEOs and XAPEOs in positive ionization (PI) mode were those corresponding to $[M+Na]^+$. Ions monitored for progestogens were m/z 321 (NOR), m/z 335 (LEV), and m/z 337 (PROG).

APs and APECs and corresponding halogenated derivatives were detected under negative ionization (NI) conditions as $[M-H]^-$, and for $AP_1ECs$ and $AP_2ECs$ confirmation fragment ions (at the fragmentor voltage of 100 V) corresponded to $[M-CH_2COOH]^-$ and $[M-CH_2CH_2OCH_2COOH]^-$, respectively. Steroid sex hormones detected under NI conditions were E1 (m/z 269), E2 (m/z 271), E3 (m/z 287), EE (m/z 295), and DES (m/z 267).

## RESULTS AND DISCUSSION

In order to optimize the switching time for steps 1 and 2, numerous experiments were performed comparing performances of different ADS RAM precolumns (C4, C8, and C18) and testing different solvents for matrix elution and the analyte transfer. In an ideal situation, the precolumn packing should only retain and enrich the analyte(s) while all other sample components (unwanted matrix) are discharged to waste with the eluent. The best results in terms of recovery of target compounds and selectivity were obtained using the LiChrospher ADS C4 precolumn. It was found that for hydrophilic analytes (APECs), the C8 and C18 precolumns had an advantage with respect to long breakthrough time, but transfer of the most hydrophobic compounds (APs and XAPs) was poor, as was the removal of matrix components. From the other side, using the C4 precolumn, very good fractionation of matrix components and analytes was achieved and good transfer of hydrophobic compounds. However, the breakthrough time for the most polar compounds was short (Table 2), and therefore the fractionation step was limited to a maximum of 5 min. Recoveries (using the ADS C4 precolumn) ranged from 62 to 104% with RSD from 0.87 to 14.5%. Chromatograms showing the separation of alkylphenolic compounds and steroid sex hormones, extracted from the spiked sediments using a RAM–LC integrated system, are shown in Figs. 2 and 3, respectively. When using RAM for cleanup of sediment extracts, the analytes and hydrophobic smaller matrix molecules are adsorbed, while larger matrix molecules, such as

**TABLE 2**
**Validation Parameters of RAM–LC–MS Integrated Method Using LiChrosphere ADS-C4 Precolumn (Merck, Darmstadt, Germany)**

| Compound | Breakthrough Time (min) | Recovery (%) | RSD (%), n = 3 |
|---|---|---|---|
| $AP_1EC$ | 5.5 ($OP_1EC$) | 62 ($OP_1EC$) | 5.5 |
| | 8.0 ($NP_1EC$) | 77 ($NP_1EC$) | 2.5 |
| $AP_2EC$ | 8 ($OP_2EC$) | 94 | 0.87 |
| | 12 ($NP_2EC$) | | |
| $XAP_1EC$ | <15 | 96 | 3.2 |
| $XAP_2EC$ | <15 | 97 | 4.1 |
| APEOs | <15 | 96 | 2.8 |
| XAPEOs | <15 | 97 | 3.1 |
| APs | <15 | 102 (OP) | 8.5 (OP) |
| | | 99 (NP) | 8.8 (NP) |
| XAPs | <15 | 83 (ClNP) | 11.8 (ClNP) |
| | | 81 (BrNP) | 14.5 (BrNP) |
| E1 | <15 | 104 | 3.6 |
| E2 | <15 | 101 | 2.5 |
| E3 | 10 | 94 | 2.5 |
| EE | <15 | 98 | 7.1 |
| DES | <15 | 97 | 5.7 |
| NOR | <15 | 96 | 2.3 |
| LEV | <15 | 103 | 4.1 |
| PROG | <15 | 102 | 4.0 |

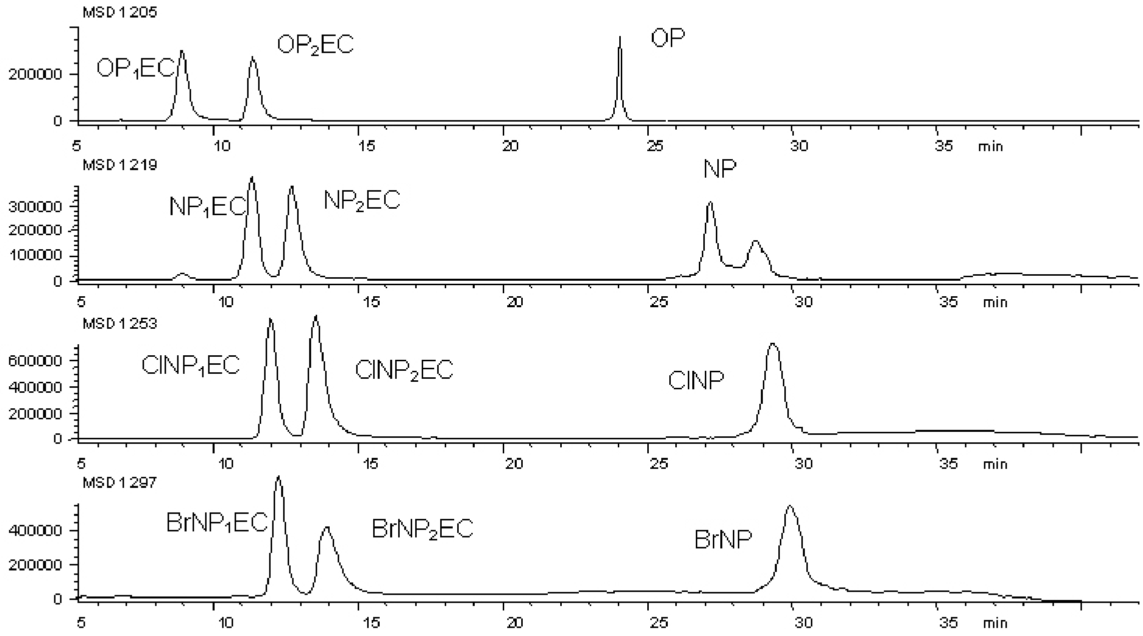

**FIGURE 2.** RAM–LC–MS extracted chromatograms for alkylphenolic compounds detected under NI conditions.

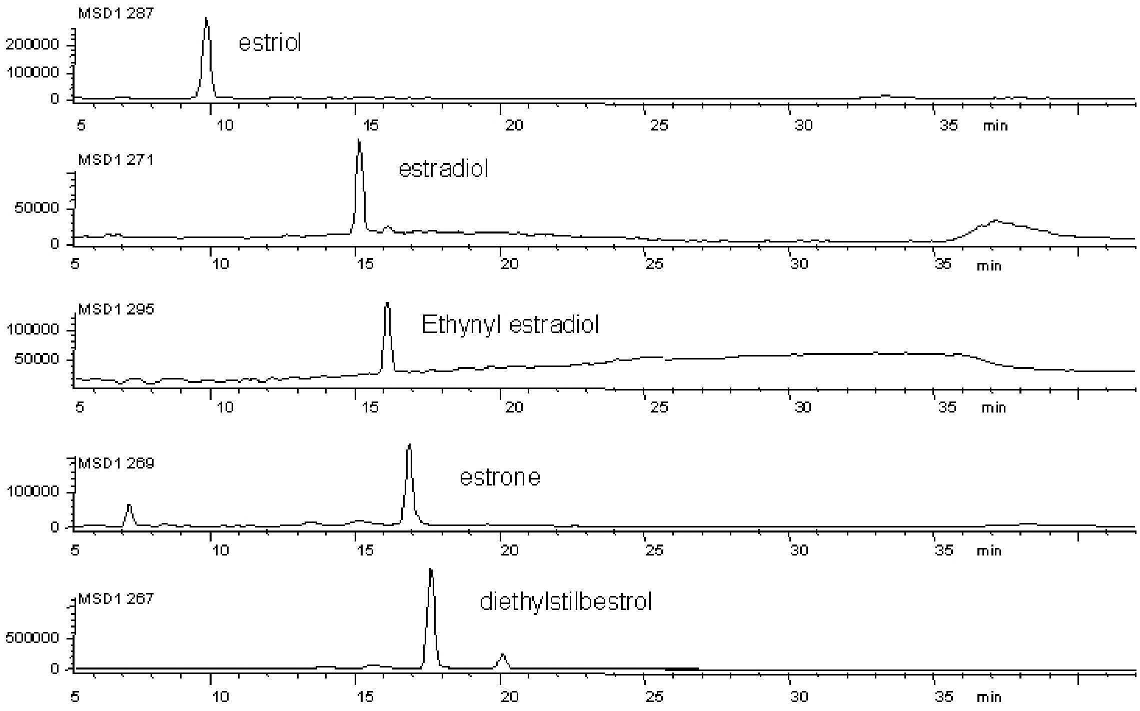

**FIGURE 3**. RAM–LC–MS extracted chromatograms for steroid sex hormones detected under NI conditions.

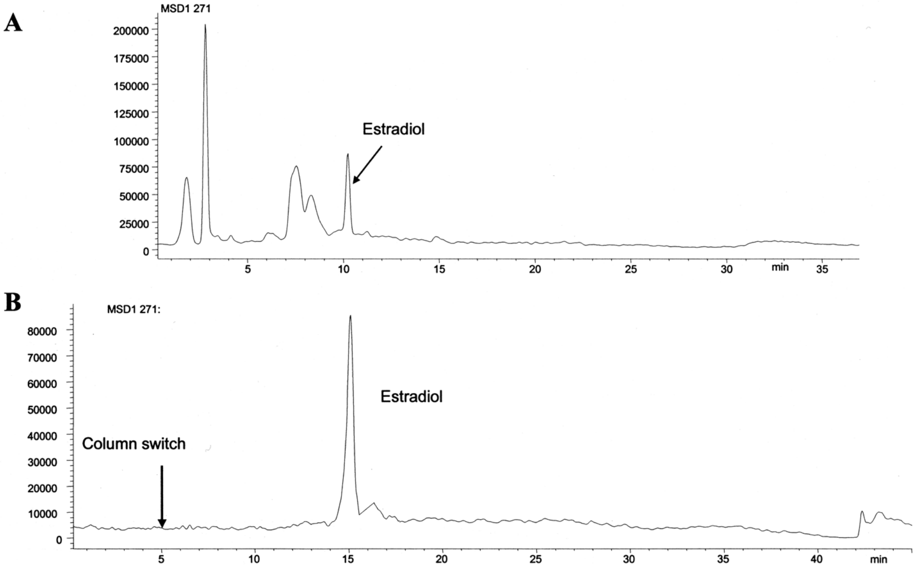

**FIGURE 4**. Comparison of LC–MS extracted chromatograms for estradiol (m/z 271) in spiked sediment: (A) ASE-SPE-LC-MS; (B) ASE-RAM (C4) LC–MS.

humic substances or proteins, as well as very polar matrix components are eluted in the dead volume of the RAM column and pumped into the waste. The efficiency of the cleanup process is shown in Fig. 4. The elimination of interfering polar matrix components led to more sensitive detection of estradiol. Generally, limits of detection (LODs) achieved using RAM–LC were approximately one order of magnitude lower than those achieved using SPE cleanup.

## ACKNOWLEDGEMENT

This paper was presented at the CSIC/ESF workshop Analysis, Toxicity, and Biodegradation of Organic Pollutants in Groundwater from Contaminated Land, Landfills, and Sediments, Barcelona, Spain, November 8–10, 2001.

This work has been supported by the EU projects from the Environment and Climate Programme (4th Framework Program) and Sustainable Development Programme, Key Action Sustainable Management and Quality of Water (5th Framework Program): SANDRINE (ENV4-CT98-0801) and SEDNET Network (EVK-2001-00058).
